# Supplementary figures and images for: Cell-Autonomous and Non-Cell-Autonomous Mechanisms Concomitantly Regulate the Early Developmental Pattern in the Kelp Saccharina latissima Embryo
Source: Plants (Basel). 2024 May 13;13(10):1341. doi: 10.3390/plants13101341 (PMC11125204; doi:10.3390/plants13101341)

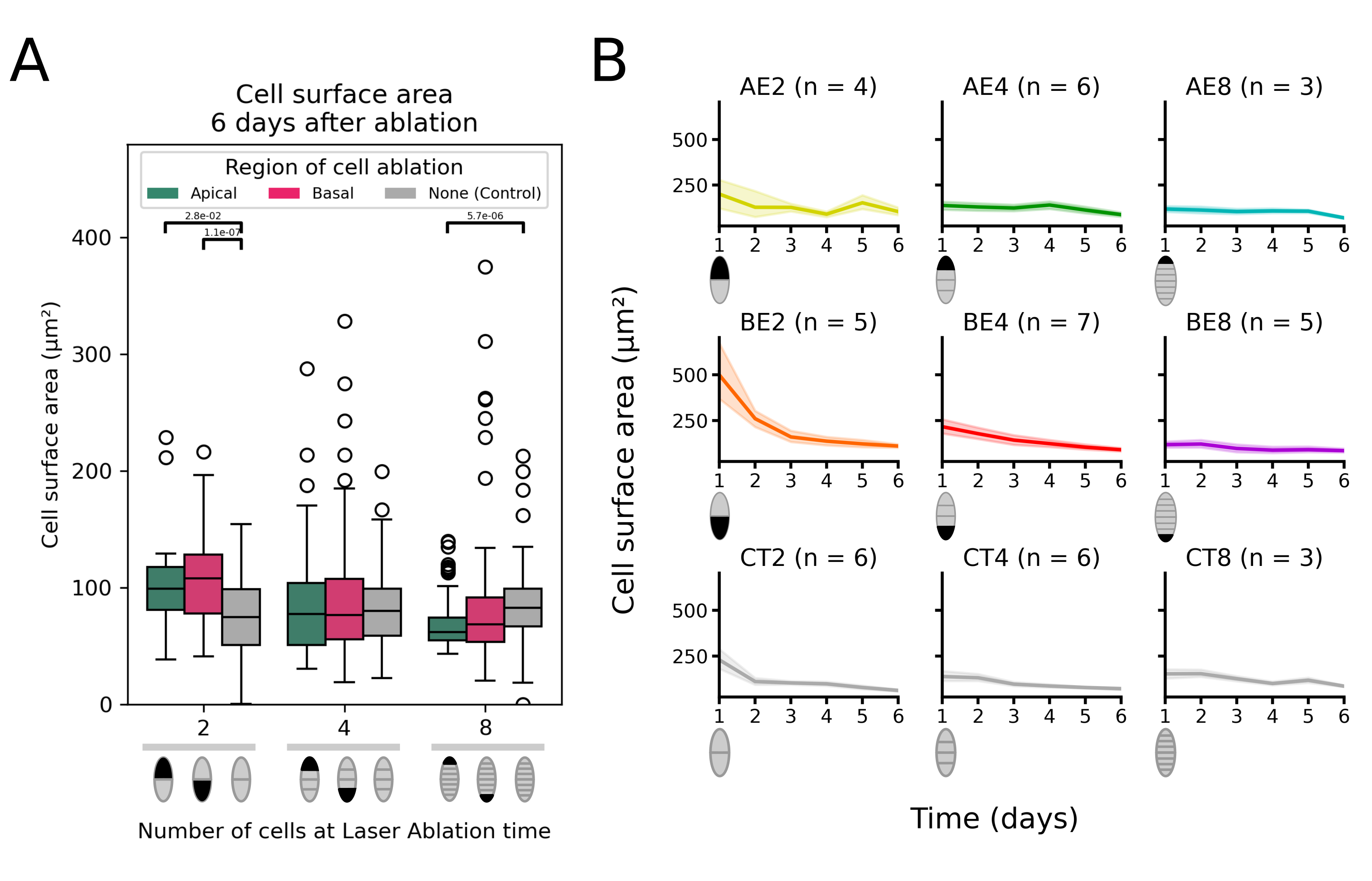

Supplement: Supplementary file 1 [file plants-13-01341-s001.zip › Fig_S1_R1.png]

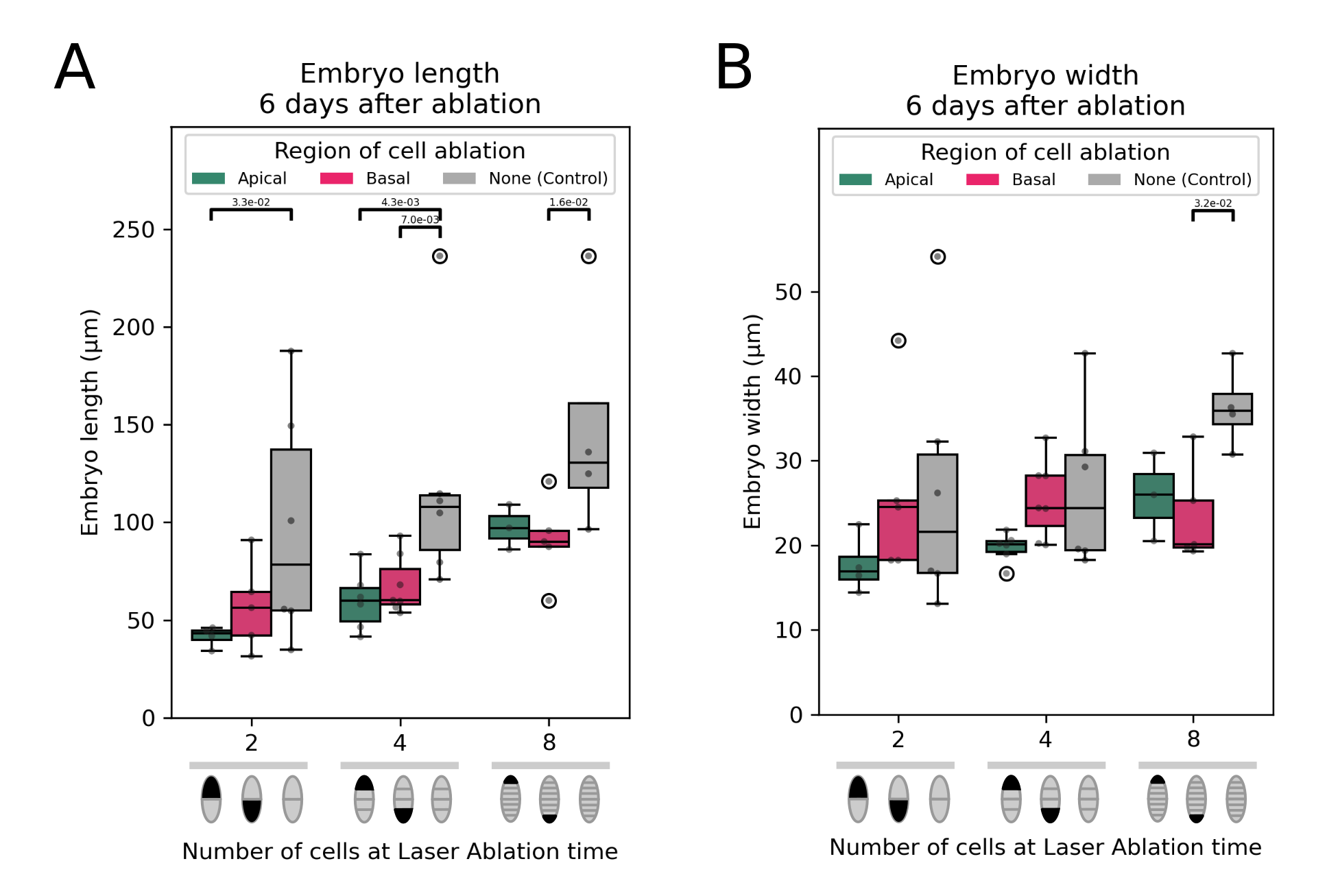

Supplement: Supplementary file 1 [file plants-13-01341-s001.zip › Fig_S2_R1.png]

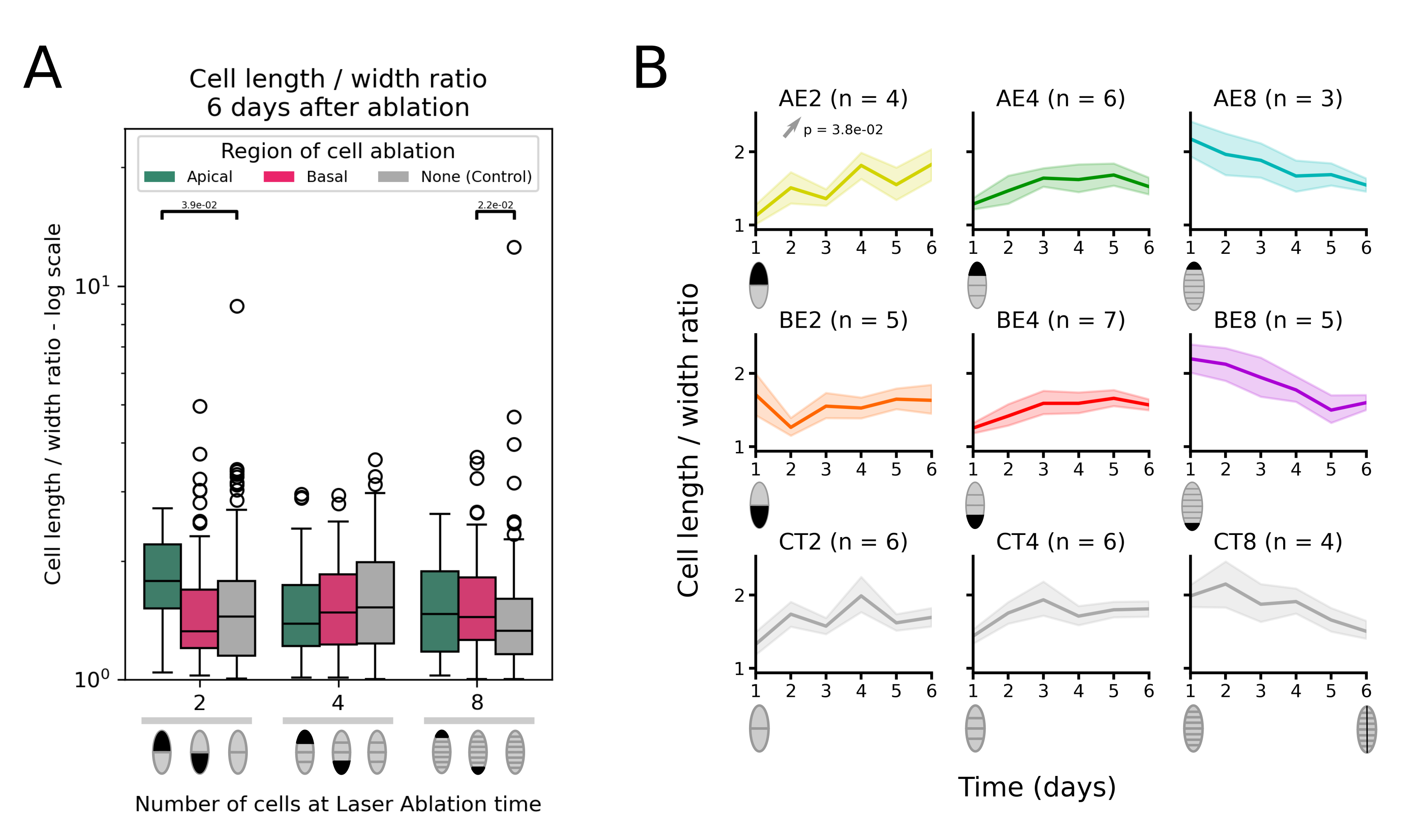

Supplement: Supplementary file 1 [file plants-13-01341-s001.zip › Fig_S3_R1.png]
